# Infantile fetiform abdominal mass: Teratoma or fetus in fetu? A case report with insights into radiological diagnosis and surgical management

**DOI:** 10.1016/j.radcr.2023.12.031

**Published:** 2024-01-13

**Authors:** Jitendra Sharma, Upasna Yadav, Varun Tej, Rajesh Malik, Radha Sarawagi, Nadeem Rahman, Aman Kumar, Ankur Patel, Abhinav C. Bhagat

**Affiliations:** Department of Radiodiagnosis, AIIMS Bhopal, Bhopal, Madhya Pradesh, India

**Keywords:** Fetus, Teratoma, CT scan, X-ray, Ultrasonography, Tumor markers, Child, Abdomen, Laparotomy

## Abstract

Fetus-in-fetu (FIF) is a rare congenital anomaly in which a malformed parasitic twin develops within the body of a live fetus or child. Abdominal teratoma, a type of germ cell tumor, can be a great imaging mimicker of FIF and vice-versa, as they both can present as a heterogeneous mass with calcifications and a fat component. Radiological differentiation of these 2 entities should be made because of the difference in surgical planning and treatment options. Features such as visualization of distinct bony vertebral elements and encysted cystic components are the specific features of Fetus in fetu [1]. In contrast, the presence of elevated serum markers can help diagnose teratoma. Here, we report a case of a 5-month-old girl presented with progressive distension of the upper abdomen for the last 2 months, noticed by her mother. Her initial imaging with abdominal X-ray and ultrasonography showed the presence of a large heterogenous solid-cystic mass in the upper abdomen with large elongated calcifications. A provisional diagnosis of teratoma vs FIF was considered. CECT abdomen showed clear identification of osseous structures of the axial and appendicular skeleton within a fat density mass, along with an encapsulated cystic component, strongly suggestive of FIF. Her serum tumor markers were within normal limits. The final diagnosis of FIF was confirmed on Laparotomy and postoperative specimens.

## Introduction

Fetus in fetu (FIF) is a very rare congenital malformation while Abdominal Teratoma, on the other hand, is a type of germ cell neoplasm consisting of at least 2 of 3 germ layers, that is , endoderm, mesoderm, and ectoderm. The abdominal cavity, particularly the retroperitoneum, is a common location for the development of teratoma.

Both FIF and abdominal teratoma can have similar clinical presentation as progressive abdominal swelling and distension. Imaging-wise, these can also mimic each other as a fetiform mass with variable amounts of fat and bone-like calcifications. Both require surgical intervention, albeit with different surgical approaches and overall management strategies. Since FIF is a benign malformed tissue, chances of malignant transformation and postoperative recurrence are very low, and simple surgical resection of mass is sufficient in most cases, with no further follow-up required. On the other hand, teratomas being a true neoplasm, have chances of malignant transformation and require a complete staging before surgery, including tissue biopsy and serum tumor markers evaluation such as serum Alfa feto protein (AFP) and beta-human chorionic gonadotropin (HCG). Management of teratomas may also include chemotherapy before or after surgery, depending upon the stage of the tumor and other clinical factors [2].

Hence, a clear radiological differentiation is necessary before surgery. This case report highlights the radiological workup of fetiform mass in an infant with emphasis on radiological differentiation between FIF and Teratomas and surgical approach towards FIF.

## Case report

A 5-month-old girl presented to the pediatric surgery department with complaints of distension of the upper abdomen for the last 2 months, as noticed by her mother. It was gradually increasing and not associated with pain. There was no history of any gastrointestinal or genitourinary symptoms. Her birth history was normal. No history of maternal illness or exposure to radiation or drug intake could be found during pregnancy. There was no previous history of twin pregnancy in the mother.

On examination, there was a focal bulge in the upper abdomen in the left hypochondrium and epigastrium region, which was hard on palpation. The child was referred to the radiology unit for further evaluation.

**Fig. 1:**
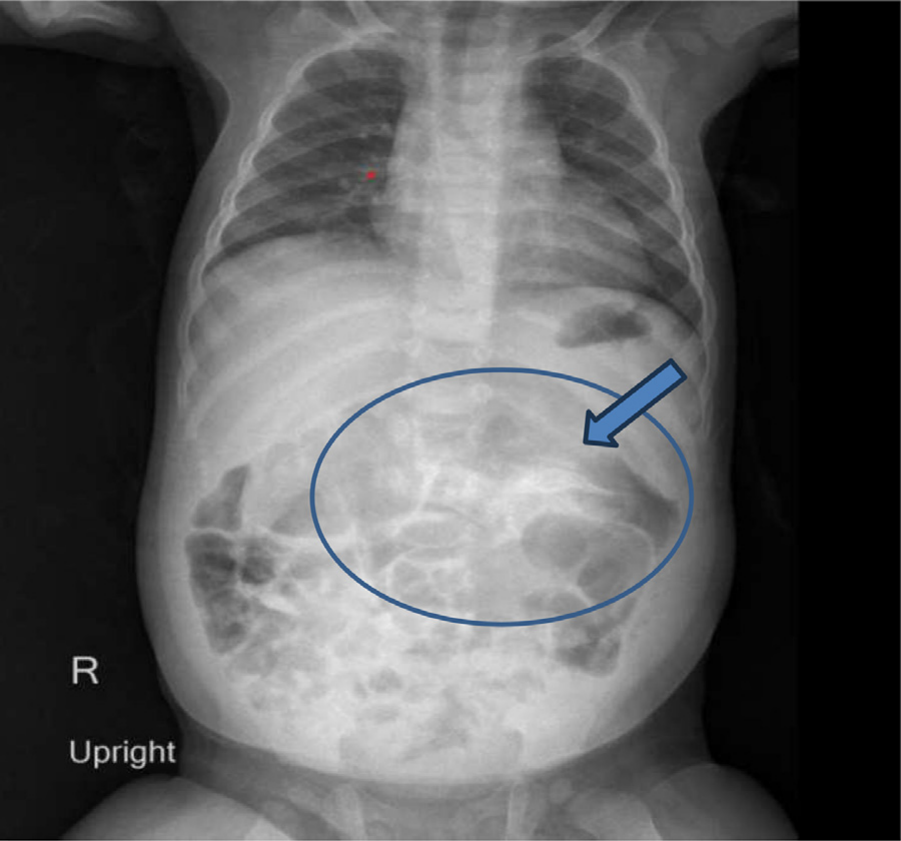
*AP Chest and abdominal X-ray* shows curvilinear bony structure (arrow) in the mid abdomen crossing the midline, resembling roughly as axial skeleton. No evidence of abnormal dilatation of bowel loops.

**Fig. 2:**
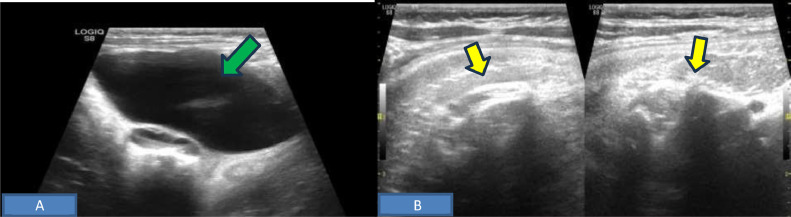
*Abdominal ultrasound* demonstrates complex lesion in the left hypochondrium with cystic component (A) and 2 echogenic line casting dense posterior acoustic shadowing and (B) possibly due to fetal spine.

Her initial evaluation with an X-ray abdomen (Fig. 1) showed a faintly visualized curvilinear bony structure in the mid-abdomen across the midline, resembling roughly an axial skeleton. On sonography, a large heterogenous solid cystic mass was seen in the upper abdomen, which predominately showed echogenic content without posterior acoustic shadowing (possibly fatty tissue), linear echogenic structure casting posterior acoustic shadowing (suggesting calcified component) and an encapsulated eccentric cystic component (Figs. 2A and B). A suspicion of infantile teratoma was given with differential of Fetus in fetu.

**Fig. 3:**
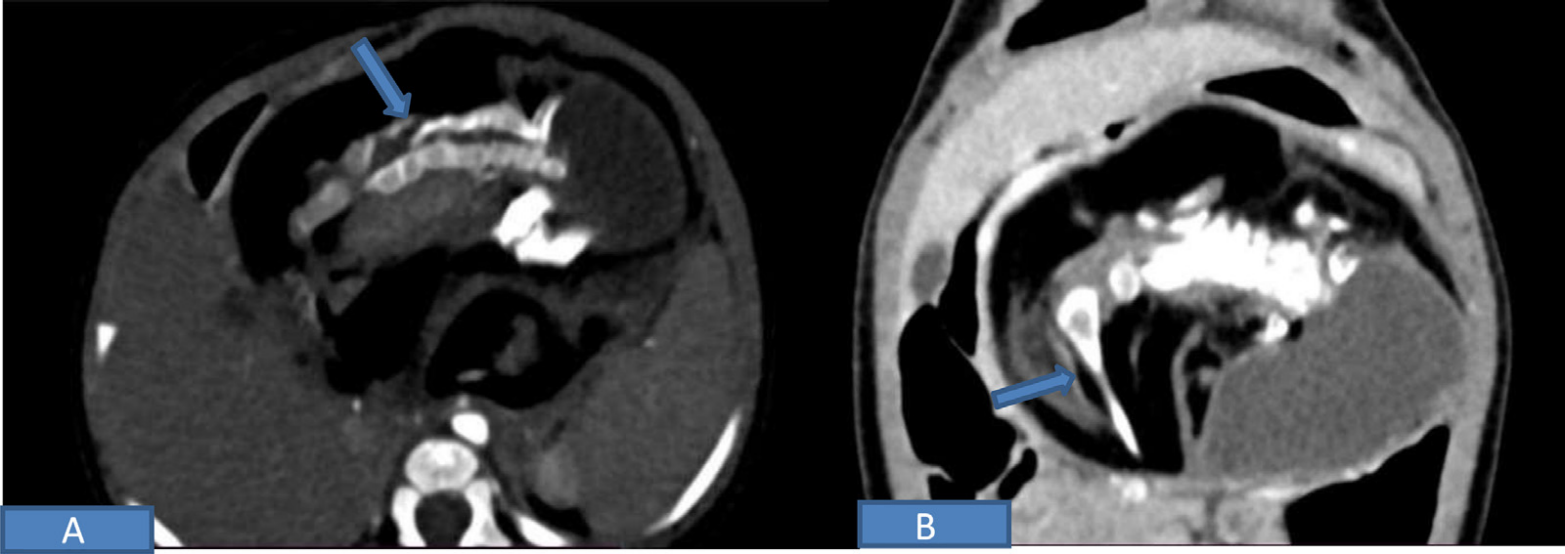
*Contrast enhanced CT image* demonstrates heterogeneous mass containing fat and calcified content. (A) axial section shows fat attenuation in lesion with well differentiated bones of vertebral column and (B) shows coronal reconstruction with the arrow's points to the corticated limb bone.

Contrast enhanced CT abdomen (Figs. 3A and B) was used to characterize mass further, revealing a large heterogeneous mass of 7.1 cm x 10.2 × 7.9 cm in the retroperitoneum. The mass had predominantly low-density fat attenuation tissue with smaller eccentric encysted fluid attenuation content. An elongated structure, consisting of a well-formed bony skeleton and surrounding adhered soft tissue, can be seen embedded within the fat tissue of the mass, giving a fetus-like or “fetiform” appearance. This fetiform tissue had multiple well-corticated osseous structures with clear identification of vertebral bodies and posterior elements, bilateral ribs, upper and lower limb bones, pelvic girdle, and shoulder girdle. The postcontrast scan showed some enhancement at the periphery and in the tissue surrounding the Fetus, with a predominant supply from splenic artery branches. The mass was displacing the pancreas anteriorly and IVC posteriorly. Surrounding structures were displaced by mass, however uninvolved. Her Serum AFP levels were also within normal limits (63 pg/mL). Based on these findings, the final presurgical diagnosis of fetus in fetu was considered.

**Fig. 4:**
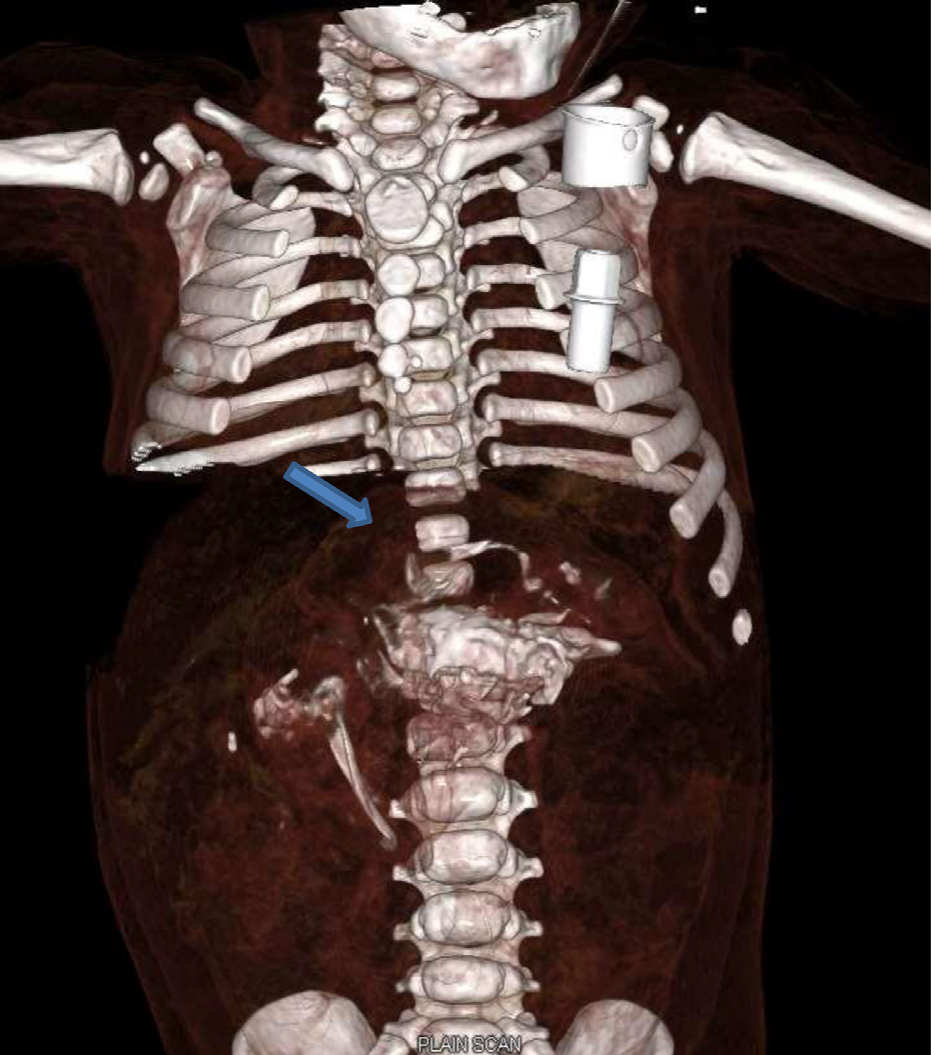
*Volume rendered imaging* of the abdomen shows 3D image of the fetus in fetu mass.

Because of progressive abdominal distension, exploratory Laparotomy was planned with the intention of gross in toto resection of the lesion. Three-dimensional volume rendered images were also created (Figs. 4A and B), which showed a better delineation of the FIF and helped pediatric surgeons plan the most appropriate surgical approach. CECT also showed a closely adhered splenic artery at the posterior aspect of the lesion; hence, the option of splenic artery ligation and splenectomy was also kept if required during surgery.

**Fig. 5:**
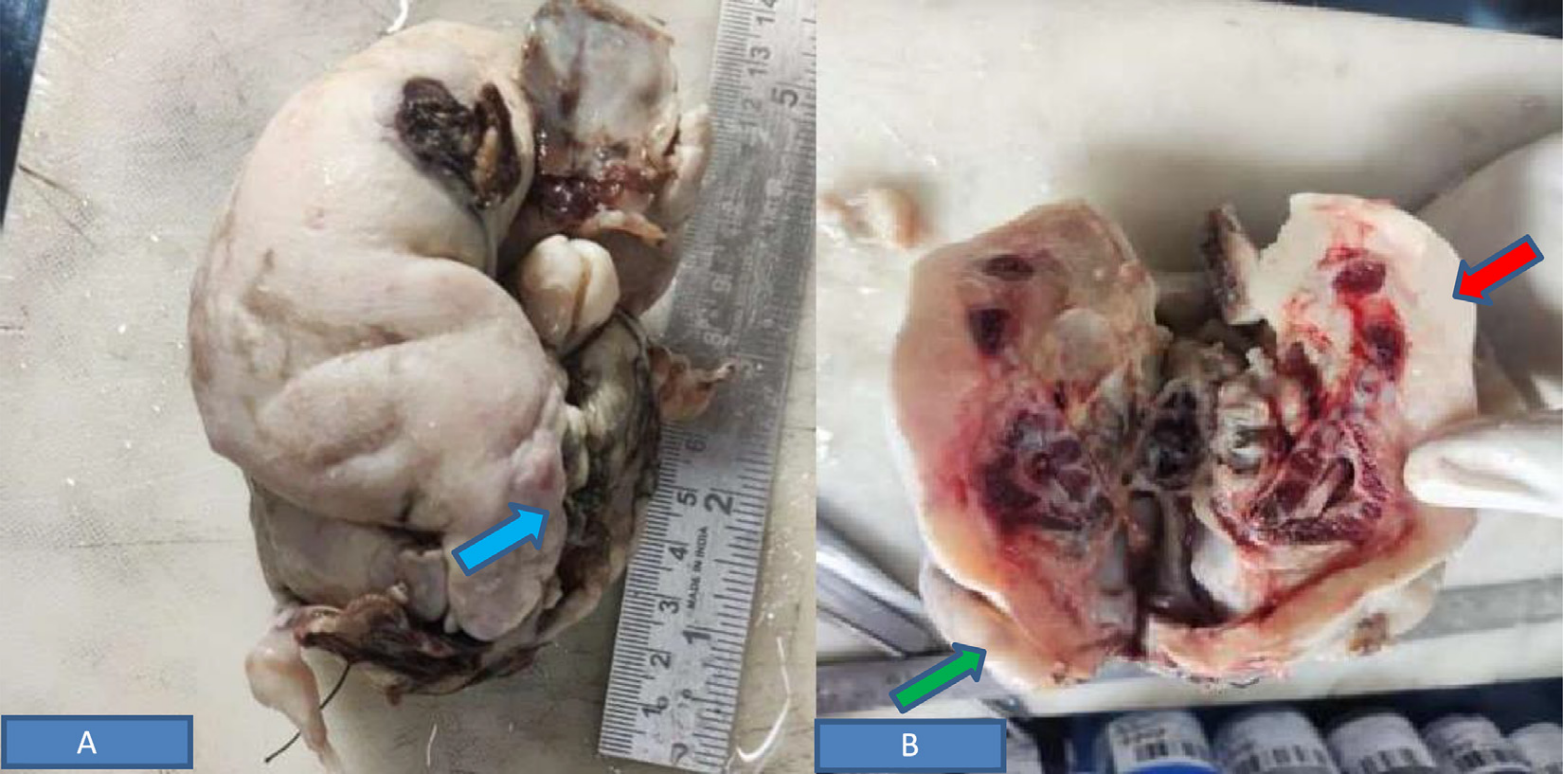
*Photos of gross specimens* (A) retroperitoneal mass covered with sac with overlying hairs (blue arrow) and (B) Specimen showing spine (red arrow) and limb buds (green arrow).

The patient underwent a Laparotomy, which revealed an encapsulated mass measuring 11 × 8 × 6.5cm, showing grossly 4 limbs, cranial vault with hair, spine (Figs. 5A and B). The cranial end shows attached membranes. This mass was covered by skin with lanugo-like hair. Grossly, the mass appears like a small fetus. The splenic artery could be separated free from the mass; hence, splenectomy was not done. On the cut section of the surgical specimen, a central meshwork of tubular structures, likely intestine, could be seen. A C-shaped spine-like structure with a 2.5-3 cm length was identified, suggesting a vertebral column. There was a cystic cavity on the cranial end extending towards both sides. Gross and microscopy confirm brain tissue, bone tissue (vertebral column), cartilage, and gastrointestinal tract. However, no definite heart, liver, kidney, or spleen were seen. No definite areas of tumoral tissue could be seen.

The child recovered well from surgery with an uneventful postoperative period. During follow-up, the child had 1 episode of vomiting after 3 months of surgery, for which USG revealed no evidence of any residual/ recurrent lesion. The child responded to conservative management and was discharged. After that, the patient has remained asymptomatic in clinical follow-ups for 6 months, which have been done till now.

## Discussion

Fetus in fetu is a rare congenital developmental disorder with an incidence of 1 in 5,00,000 births [3]. This happens when a malformed parasite twin gets entrapped within the body of its host twin and receives blood supply from host. The term was first coined by Willis [4]. Multiple theories have been postulated in the pathogenesis of FIF; the most widely accepted theories include aberration in the embryogenesis in monozygotic diamniotic twinning in which one of the fetus is enclosed within the host during the ventral folding of trilaminar embryonic disc [5]. Another accepted theory suggests that it belongs to the teratoma spectrum, with FIF being a highly organized form of teratoma.

The main entity to be differentiated from FIF is organized teratoma. According to Willis, an axial skeleton with a vertebral column is an important criterion for distinguishing between FIF and teratoma [4]. FIF typically originates from a zygote at the primitive streak stage. The presence of vertebral elements indicates that the stage of gastrulation has already been completed since the fetiform mass develops to a degree akin to normal fetal development [6].

The initial growth of FIF is similar to that of twins, yet it ends suddenly for 1 of 2 reasons: either the vascular superiority of the host twin or an inherent pathology in the parasite twin. The most common location of FIF is retroperitoneum (80%) [7,8]. Other rare locations such as the oral cavity, scrotum [9], and sacrococcygeal region [1] are also noted.

FIF is usually asymptomatic and produces symptoms when it is large enough to exert a mass effect. Anencephaly is seen in almost all cases of FIF, but in our case, there is a cranial vault with definite brain tissue. The common symptoms are abdominal distension, vomiting, hydronephrosis, jaundice, and respiratory distress.

Radiological imaging plays a crucial role in the preoperative noninvasive diagnosis of this entity with utmost certainty. The plain X-ray is helpful in the initial evaluation of abdominal distension and to rule out other causes such as bowel obstruction, pneumoperitoneum, etc. When skeletal features in the form of limb bones and spinal columns are detected, abdominal radiographs are sufficient.

Sonography also plays an important role in identifying the solid-cystic nature of the mass.

The main investigation of choice is contrast-enhanced CT, which is helpful in identifying the exact location, fetiform structures, vertebral organization, malignant transformation, vascular supply, and relations with adjacent structures and for deciding surgical planning. In our case, a contrast-enhanced computed tomography (CECT) of the abdomen was performed to delineate the mass and assess the organ of origin clearly.

Underlying a central bony feature on CT scans of patients with FIF is a mass shaped like a spherical or tubular collection of fat. CT aids in pinpointing the precise link between FIF and the nearby structures as well as the displacement of those structures as a result of the mass effect. In our example, a detailed depiction of bone structures was achieved by the use of volume rendering techniques in three-dimensional CT scans [10].

The differentiation between FIF and organized teratoma is crucial, as teratoma has more potential for malignant degeneration than FIF and needs close follow-up with sonography and AFP serology. The most often used serum marker for teratoma diagnosis and follow-up is the AFP level in the serum. The source of AFP is the fetal liver, although, during the early stages of fetal development, the yolk sac is also actively involved in the synthesis of AFP. AFP is present throughout pregnancy and is a useful indicator of malignancies generated from the yolk sac and embryos. The degree of early malignancy may be correlated with the absolute amount of blood AFP, and tracking trends in serum AFP levels can be helpful in tracking tumor recurrence following resection. B-human chorionic gonadotropin (Beta HCG) and, less frequently, carcinoembryonic antigen (CEA) have been used as malignancy indicators for gonadal teratoma [1].

Whether FIF is a distinct organism or a well-organized teratoma is still a matter of debate. A teratoma is a potentially malignant tumor that comprises numerous tissues from all 3 germ layers. For this reason, some authors refer to FIF as a well-structured teratoma. According to a few authors, fetiform teratomas and conjoined twins are at 1 extreme of the continuum of abnormalities that might arise from aberrant embryogenesis in a monochorionic pregnancy; in between are parasitic twins and embryonic vestigial fetal inclusions [10].

There are certain differences between teratoma and FIF entity. Teratomas are morphologically classified into extra-gonadal (80%) and gonadal (20%) locations, and they typically develop in the midline at any level of the body. The pharynx or palate, in the sphenoid region known as the Rathke pouch, is the site of epicanthus or oropharyngeal teratoma. In contrast to teratoma, cases of fetus in fetu have been documented in the literature, with the retroperitoneal site being the most common [6].

Abdominal teratomas are extremely rare. Teratomas may or may not be linked to other abnormalities. For example, presacral mass, anal stenosis, and sacral deformities are connected with autosomal dominant Currarino Triad. No such association or syndrome has been identified in FIF cases yet. Surgical excision is the treatment of choice as FIF also carries a small risk of malignant degeneration due to the presence of immature elements. There is only 1 documented case of malignant transformation in FIF in the literature [11].

One of the differences also lies in the management of teratoma and that of FIF. In order to minimize chances of recurrence and depending upon other clinical factors, appropriate treatment usually involves 1 of the following [2]:

• Surgical resection followed by careful monitoring for disease recurrence
• Initial surgical resection followed by platinum-based chemotherapy
• Diagnostic tumor biopsy and preoperative platinum-based chemotherapy followed by definitive tumor resection

When combined with chemotherapy and total tumor removal in malignant situations, surgery for malignant teratoma is safe, has a low risk of complications, and has good long-term results [6].

However, in FIF gross in toto excision with meticulous dissection of the surrounding structures is to be done to avoid injury to the adjacent structures such as vessels and biliary system. Its weight and size vary according to the blood supply. FIFs with vascular connections to the host are larger and possess more advanced features. In our case, the sac was in contact with the liver and pancreas and closely related to CBD, portal vein, IVC, and aorta. The pancreas was severely displaced and compressed, with its head located antero-laterally and its body and tail antero-superiorly. The pancreas was thinned out with indistinct fat planes with the mass lesion; however, uniform homogenous enhancement was seen. The splenic artery was also closely adhered to the mass at the posterior aspect. Preoperative reporting of this information helped the surgeon regarding approach and excision.

## Conclusion

FIF is an interesting and very uncommon entity, encountered rarely once or twice in the physician and radiologist lifetime and can be confused with abdominal teratoma, particularly for radiologists. However, the cross-sectional imaging features can be very typical and can provide accurate, noninvasive, preoperative diagnosis. Identification of fetiform pattern in a teratoma-like mass that is, heterogenous mass with calcification, fat, and cystic areas, should raise suspicion of FIF. Accurate diagnosis of FIF mostly results in complete excision of the mass, which is curative.

## Patient consent

Its hereby stated that an informed consent was obtained from the guardians of this patient, regarding usage of patient diagnostic images and related clinical information for publication purpose. Patient identity had been kept confidential in this manuscript and would not be revealed at any time during or after publication.
